# A Pilot Study of Telerobotic Radical Thyroidectomy for Thyroid Cancer Using a 5G Network

**DOI:** 10.3390/jcm15103591

**Published:** 2026-05-08

**Authors:** Bing Wang, Chen Li, Zheng Wan, Jian Zhu, Meng Wang, Yanbing Jian, Zelong Yang, Xin Miao, Linlin Zhang, Fei Kuang, Lin Liu, Guolou Li, Qingqing He, Jing Yao, Wen Tian

**Affiliations:** 1 Senior Department of General Surgery, the First Medical Center of Chinese PLA General Hospital, Beijing 100853, China; 2 Department of Thyroid and Breast Surgery, the 960th Hospital of PLA Joint Logistics Support Force, Jinan 250031, China; 3 Department of Breast and Thyroid Surgery, Weifang Hospital of Traditional Chinese Medicine, Weifang 261041, China; 4 School of Medicine, Nankai University, Tianjin 300071, China

**Keywords:** thyroid cancer, thyroidectomy, 5G communication technology, remote surgery, robot surgery, mobile healthcare

## Abstract

**Background**: The incidence of thyroid cancer has increased globally. In recent years, robotic surgical systems have been applied in thyroid surgery, and the rapid development of fifth-generation (5G) communication technology has laid a solid foundation for the smooth implementation of remote surgery. **Objective**: The aim was to explore the feasibility and safety of telerobotic radical thyroidectomy using 5G communication technology to treat thyroid cancer. **Methods**: From August 2024 to October 2024, telerobotic radical thyroidectomy was performed on seven female patients using a 5G wireless network and a dedicated line network (or ordinary wired broadband) spanning 22–2200 km. The patients’ clinical and information transmission data were analyzed. **Results**: All patients (papillary thyroid carcinoma, female, with an average age of 44.0 ± 4.6 years) underwent uneventful surgical procedures without any transfer to open surgery or complications. The average surgical duration was 91.3 ± 11.8 min, the average blood loss was 11.4 ± 4.8 mL, and the average postoperative hospital stay was 3.6 ± 0.8 days. All subjects were successfully discharged within 5 days after surgery. The average total latency time of the intraoperative network was 137.5 (range, 121–159) ms, and there were no adverse events, such as network disconnection, frame loss, or network attacks. The operator worked smoothly without any obvious delay or lag, and the recorded audio and video are clear. **Conclusions**: Telerobotic radical thyroidectomy for thyroid cancer over a 5G network demonstrates promising feasibility and safety. With stable network transmission and a clear surgical field, the precise operations required in thyroid surgery can be performed reliably. These findings suggest that this technology can facilitate high-quality surgical care in remote areas, contributing to a more balanced distribution of medical resources.

## 1. Introduction

The incidence of thyroid cancer, the most common endocrine malignancy of the head and neck, is rapidly increasing worldwide [1]. In China, approximately 466,100 new cases of thyroid cancer were diagnosed in 2022, accounting for about 9.7% of all malignant tumors. With thyroid cancer now ranking third among all newly diagnosed malignancies, it has become a health threat that cannot be ignored [2].

Surgery is the most effective curative method and first line of management for thyroid cancer. In recent years, the rapid development of robotic surgical technology has demonstrated significant advantages for thyroid surgery. A robotic surgical system can provide a three-dimensional field of vision and the ability to perform fine operations with multiple degrees of freedom, helping surgeons accurately identify and separate important anatomical structures, such as blood vessels and nerves, and perform delicate operations such as suturing, releasing, and cutting within a smaller surgical space [3]. Compared with traditional surgical methods, robotic thyroid surgery offers benefits such as improved esthetic outcomes, less intraoperative bleeding, faster postoperative recovery, and a lower incidence of complications, greatly improving surgical safety and patient satisfaction [4].

The uneven distribution of medical resources and significant differences in medical service levels between urban and rural areas in China make it difficult for some thyroid cancer patients to receive high-quality diagnosis and treatment services. However, the use of robotic systems in thyroid surgery is becoming increasingly common. Research on remote robotic surgery began as early as 30 years ago, when doctors in the United States and the United Kingdom began to perform the procedure [5,6]. Recently, an international multispecialty consensus statement and expert opinion on best practices in telesurgery was published [7]. The emergence and development of remote surgical technology based on robotic surgical systems have overcome spatial barriers and geographical limitations, expanded the coverage of high-quality medical resources to benefit patients in remote areas with limited access to medical resources, and effectively improved the unequal distribution of medical services [8,9]. The rapid development of 5G communication technology has played a crucial role in this technological innovation. Characterized by its low latency, high speed, wide connectivity, and high reliability, 5G technology enables the high-quality transmission of three-dimensional images, addresses traditional network latency and transmission quality issues, ensures real-time and accurate data transmission during surgical procedures, and lays a solid foundation for the smooth implementation of remote surgery [10].

In this study, we present telerobotic radical thyroidectomy using 5G communication technology to treat thyroid cancer and evaluate its feasibility and safety.

## 2. Methods

### 2.1. Study Population and Surgical Team

A total of seven patients (aged 18–70 years) who underwent 5G-facilitated telerobotic radical thyroidectomy for thyroid cancer between August 2024 and October 2024 were enrolled in this pilot study according to the inclusion and exclusion criteria (outlined in the Clinical Practice Guidelines for Robot-Assisted Thyroid and Parathyroid Surgery—2024 Edition [11]). The inclusion criteria were as follows: (1) Primary surgery for differentiated thyroid carcinoma (DTC), with a tumor maximum diameter ≤ 2 cm; no invasion into adjacent structures, including the trachea, esophagus, larynx, or major blood vessels and nerves; no extensive cervical lymph node metastasis or fusion, adhesion, or fixation of enlarged lymph nodes; and no upper mediastinal lymph node metastasis or distant metastasis. (2) Willingness to undergo remote robotic thyroid surgery and provision of written informed consent. The exclusion criteria were as follows: (1) Presence of severe concomitant diseases rendering the patient unable to tolerate surgery. (2) History of prior neck surgery or radiotherapy. (3) Tumors with a maximum diameter > 2 cm. (4) Differentiated thyroid carcinoma—tumors with extrathyroidal extension involving the esophagus, trachea, larynx, or major blood vessels; extensive cervical lymph node metastasis with cystic degeneration, or fused, adherent, and fixed lymph nodes; and presence of upper mediastinal lymph node metastasis or distant metastasis. (5) Pregnancy. (6) Preoperative cytology confirming high-risk or unfavorable pathological subtypes (anaplastic carcinoma; poorly differentiated thyroid carcinoma). (7) Inability of the network infrastructure to guarantee a stable, low-latency fiber optic connection. (8) Refusal to undergo remote robotic thyroid surgery. This study was approved by the Ethics Committee of PLA General Hospital (protocol code: S2024-488-01; approval date: 30 July 2024), and written informed consent was obtained from all patients. To prevent security risks during the surgical process caused by network signal issues, a specialist was present in close proximity to ensure patient safety and handle any unexpected situations. The lead surgeon was a board-certified thyroid surgeon with over 10 years of experience in robotic thyroid surgery. He completed the formal training curriculum for the da Vinci Surgical System and holds a valid license for robotic surgery. Prior to this study, he had performed more than 1000 robotic thyroidectomies.

### 2.2. Remote Robotic Surgical System and Network Connections

**Figure 1:**
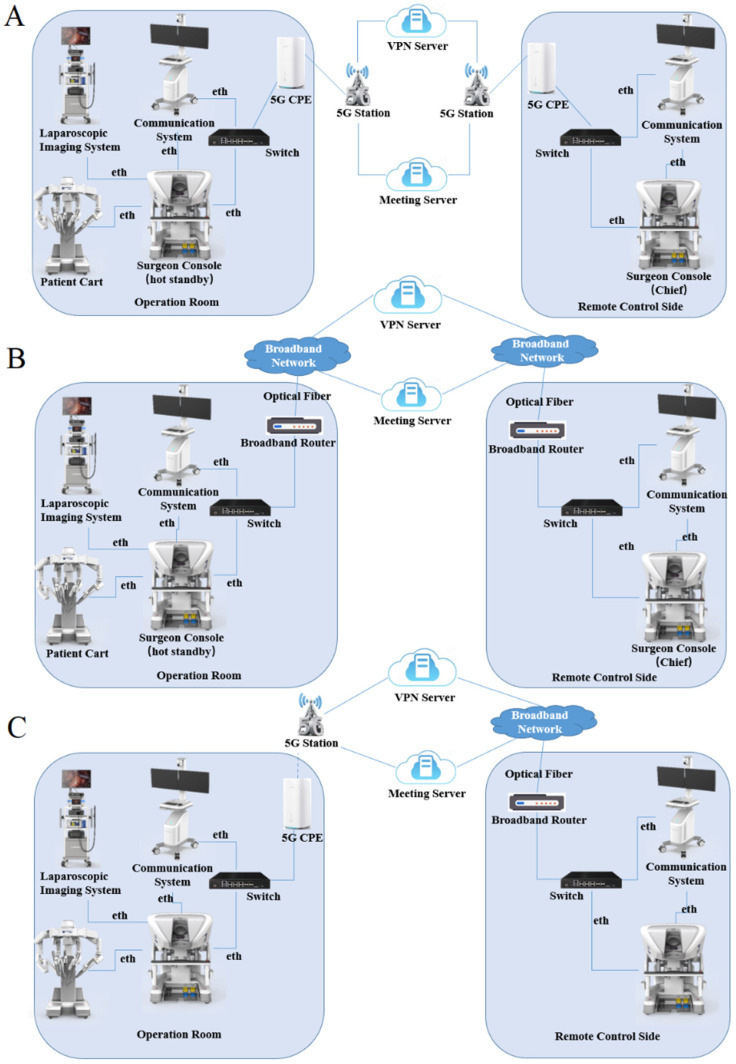
Schematic diagram of remote robotic surgical system operation and network connection. (**A**) 5G network topology; (**B**) Broadband network topology; (**C**) 5G + Broadband network topology.

**Figure 2:**
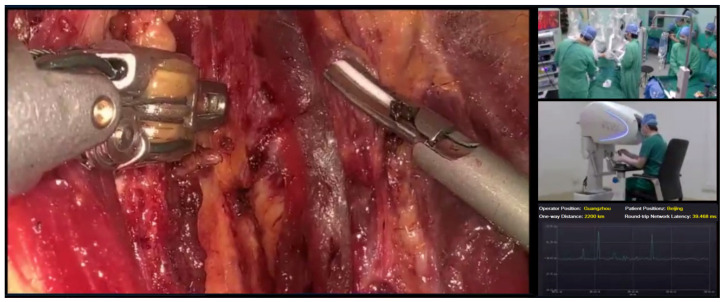
The 4K display screen of the video conference system.

Among the seven enrolled patients, the first underwent fixed-point remote robotic thyroid surgery. An MP1000 Surgical Robot System (Shenzhen Edge Medical Technology Co., Ltd., Shenzhen, China) was used. The surgeon’s console (master system) was located in the remote center of the Sun Yat Sen Memorial Hospital of Sun Yat Sen University (Guangzhou), and the console (slave system) operated by the assistant beside the bed was located in the operating room of the Third Medical Center of the PLA General Hospital (Beijing). The remaining six patients underwent mobile remote robotic surgery using a Toumai MT-1000 vehicle-mounted remote mobile surgical robot (Shanghai MicroPort MedBot (Group) Co., Ltd., Shanghai, China). In these six cases, the master systems were located in a remote mobile vehicle. In four of these cases, the corresponding slave systems were located in the operating room of the First Medical Center of the General Hospital of the People’s Liberation Army, and the remaining two cases were located in the operating room of Weifang Hospital of Traditional Chinese Medicine (the detailed locations and spanning distances are shown in Table 1). Fixed-point remote surgery uses a wireless network and a dedicated network to achieve interactive transmission of master–slave data, whereas mobile remote surgery uses a wireless network and an ordinary wired network or a wireless network plus a wireless network to achieve interactive transmission of master–slave data. In this study, the dedicated line network was a dedicated line of the public internet (100 Mb/s, China Unicom, Beijing, China), which was a 5G network (China Telecom, Beijing, China), and the IP addresses were allocated by 5G signal base stations in the public network. Figure 1 illustrates the 5G and broadband connections and their hybrid access configuration, as well as their connections to the cloud servers used. Remote robots only accept Ethernet wired connections. Any network access method that can provide an Ethernet interface and connect to our Virtual Private Network (VPN) and video conferencing servers through this network can achieve bidirectional communication. At a specific moment, a single endpoint can only use one type of network access, but two endpoints can use different types of network access. To ensure the security of surgical signal transmission, two types of networks were debugged and encrypted before surgery, and a new-generation firewall was applied to prevent network attacks. The surgery was visualized through a remote surgical demonstration system, in which the master and slave systems, as well as the surgical images, were displayed on 4K screens in two locations. The surgeon (master) and assistant (slave) communicated seamlessly through a video conferencing system, showing the surgery in both locations in real time (Figure 2). The patient’s vital signs and the surgical site conditions were also transmitted in real time through the network, allowing the surgeon to control the operation in a timely and comprehensive manner and understand the surgical dynamics. In addition, multiple adjustments and tests were conducted on the surgical robot platform and the master–slave system network before surgery to ensure a smooth procedure (supplementary technical information is provided in the Supplementary Materials).

### 2.3. Equipment Debugging and Surgical Scenarios

The robot engineering teams of both the master and slave systems turned on the equipment one hour before the surgery, performed network connection and device debugging between the master and slave systems, and monitored network latency. The on-site images of all parties, the parameter information of the anesthesia machines and monitors, and the robot surgery images were stable and clear, with real-time synchronized and clear sound.

### 2.4. Surgical Procedure

After ensuring that the equipment and network connections were in good condition, general anesthesia was administered. After the patient was anesthetized, they were placed in a supine position, with their shoulders and back elevated, head tilted back, and neck fully exposed. The upper limbs were tightly attached to both sides of the chest wall and fixed. The skin in the area routinely disinfected with iodine was covered with sterile wipes. Seven remote surgeries were performed using the bilateral axillary areolar approach (BABA). The specific operation steps were as follows: arc-shaped incisions of approximately 12 and 10 mm were made at the 1 and 11 o’clock positions of the right and left areolas, respectively. Incisions of 10 mm were made at the folds of the left and right axillary lines, and the incisions were used as injection points to inject 80 mL of swelling fluid (500 mL of physiological saline, 40 mg of ropivacaine, and 1 mg of adrenaline). A subcutaneous tunnel was established using a separation rod to separate the subcutaneous tissue along the suprasternal fossa in the superficial layer of the deep fascia. A 12 mm elongated trocar was inserted through the incision of the right areola to dock with the second arm of the robot, and a 30° lens was connected and filled with low-pressure (real-time pressure of approximately 6 mmHg) and high-flow CO_2_ gas. A 10 mm trocar was inserted into the right armpit to dock with the first arm of the robot with a specialized gripper. A 10 mm trocar was inserted into the left areola to connect the ultrasonic knife with the docking robot’s third arm, and a 10 mm trocar was inserted into the left axilla to connect the fourth arm of the docking robot with the robot’s dedicated bipolar electrocoagulation. All the instruments converged at the suprasternal fossa to finish positioning and docking the robot. The skin flap was separated along the deep surface of the cervical latissimus muscle using an ultrasonic knife to establish the operating space. The separation range extended upwards to the thyroid cartilage and laterally to the lateral edge of the sternocleidomastoid muscle. The cervical white line was opened by cutting upwards to the thyroid cartilage and downwards to the upper edge of the sternum, and the anterior cervical muscle group was separated and pulled outwards to expose the bilateral thyroid glands.

**Figure 3:**
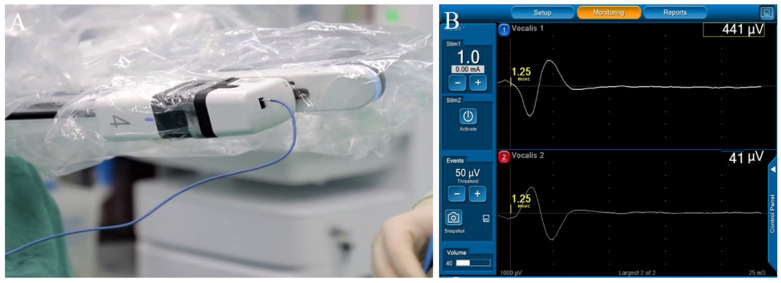
Application of intraoperative neural monitoring. (**A**) The neural monitoring probe was connected to the robotic arm; (**B**) Recurrent laryngeal nerve electromyography (EMG).

**Figure 4:**
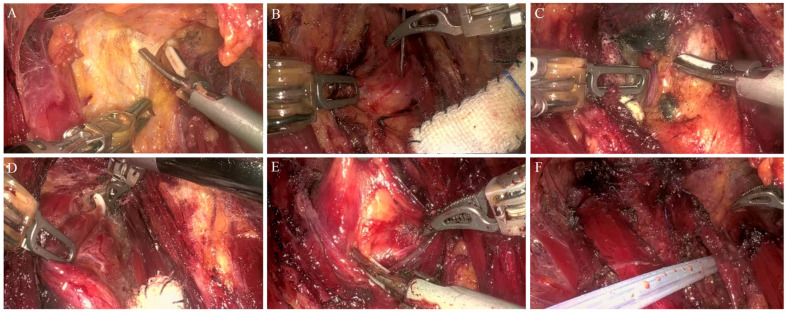
Intraoperative situation of remote robotic thyroid surgery. (**A**) Free skin flap for cavity construction; (**B**) injecting nano carbon; (**C**) expose and preserve the inferior parathyroid gland in situ; (**D**) Hem-o-lock clamp the upper pole blood vessels; (**E**) sweep the pre tracheal lymphatic adipose tissue; (**F**) indwelling drainage tube.

Different surgical scopes should be determined on the basis of the preoperative examination and intraoperative findings. A nanocarbon tracer (0.1 mL) was injected subcutaneously into the gland. After confirming the trachea, the isthmus and conical lobe were cut off to fully expose the thyroid gland lobe. A neural monitoring device was used to detect the vagus nerve and obtain V1 signals. The middle thyroid vein was cut off, the inferior pole of the thyroid gland was raised to cut off the inferior pole blood vessels, the inferior pole of the thyroid gland was separated, and the inferior parathyroid gland was exposed and preserved in situ. The cricothyroid space was separated, and a neural monitor (NIM-Response 3.0TM system, Medtronic Xomed Company, Minneapolis, MN, USA) was used to explore and determine the external branches of the superior laryngeal nerve and protect them. The upper pole of the thyroid gland was then decapitated. The superior thyroid vessels were ligated using Hem-o-lok clips (Teleflex Medical Co., Ltd., Morrisville, NC, USA) and then severed with in situ preservation of the superior parathyroid gland. A neural monitoring device was used to detect and locate the recurrent laryngeal nerve to obtain the R1 signal (Figure 3). The recurrent laryngeal nerve (RLN) was fully exposed throughout the procedure. The thyroid lobe and isthmus were finely dissected and excised. The specimen was retrieved via a bag through the No. 2 arm incision. Lymphoadipose tissue in the pretracheal and prelaryngeal areas was cleared; vessels were identified, coagulated with an ultrasonic scalpel, and transected. Lymphoadipose tissue along the ipsilateral RLN and in the tracheoesophageal groove was dissected and cleared. On the right side, special attention was paid to clearing the level VIB lymphoadipose tissue. This specimen was also bagged and removed through the No. 3 arm incision. Finally, a nerve monitoring device was used to stimulate the bilateral RLNs and vagus nerves to obtain R2 and V2 signals, confirming that bilateral neuromuscular signals were intact. Hemostasis was achieved, the wound was irrigated, and exploration confirmed no active bleeding. The cervical white line was sutured. A negative pressure suction bulb was placed in the surgical field and exteriorized through a puncture hole in the anterior axillary line, then sutured and secured. The robotic arms were undocked, all incisions were closed, and the procedure was completed (Figure 4).

### 2.5. Statistical Analysis

Data on age, average surgical duration, average blood loss, and average postoperative hospital duration are presented as means ± standard deviations (SDs). The average round-trip delay of the network, average delay of the robotic arm’s response to control signals, average delay of the surgical machine host system for image compression and decompression, and total delay are presented as the mean and range (minimum, maximum). All analyses were performed using SPSS version 23.0 software (IBM, Chicago, IL, USA).

## 3. Results

### 3.1. Demographics and Clinical Characteristics

All patients had been diagnosed with papillary thyroid carcinoma (they were female, with an average age of 44.0 ± 4.6 years). Their ACR TI-RADS grading system scores ranged from four to five. All enrolled patients underwent a preoperative FNAC, and the preoperative Bethesda grade ranged from 5 to 6, which met the indications for thyroid surgery (Table 2). Intraoperatively, the superior thyroid arteries were ligated using Hem-o-lok clips, while the inferior thyroid arteries were ablated using an ultrasonic scalpel. Intraoperative neuromonitoring was utilized in all cases to identify and preserve the recurrent laryngeal nerve. No recurrent laryngeal nerve injuries occurred. Parathyroid glands were preserved in situ whenever possible. No tracheal invasion was observed in any case.

### 3.2. Perioperative and Postoperative Pathological Condition of Patients Who Underwent Fixed-Point and Mobile Operations

All seven patients recovered smoothly after surgery without any complications, such as bleeding, hoarseness, choking on water, or symptomatic hypocalcemia. The average surgical duration was 91.3 ± 11.8 min, the average blood loss volume was 11.4 ± 4.8 mL, and the average postoperative hospital duration was 3.6 ± 0.8 days. The maximum diameter of the tumor ranged from 0.3 to 1.4 cm, with five patients (71.43%) having unilateral nodules and two patients (28.57%) having bilateral nodules. All patients were PTC and successfully discharged within five days after surgery (Table 3).

### 3.3. Intraoperative Delay and Network Security

In this study, seven cases of remote surgery were performed, of which one was performed using a 5G wireless network and dedicated network for fixed-point remote surgery. The average delay time of the bidirectional network was 39 ms, the average delay time of the robotic arm’s response to control signals was 80 ms, the average delay time of the surgical robot host system for image compression and decompression was 40 ms, and the total average delay was 159 ms. The other six cases used 5G wireless networks and ordinary wired networks in mobile remote surgery, and the detailed data are shown in Table 4; all latency times were less than 145 ms. The operator’s movements were smooth and steady, without any obvious delay or lag, and there were no incidents of network disconnection, frame loss, network attacks, or other adverse events during the surgery. The remote host system of the surgical robot successfully ensured network security using encryption technology and next-generation firewalls.

## 4. Discussion

In 2001, for the first time, Marescaux remotely controlled the French ZEUS robot from 14,000 km away in New York via high-speed fiber optic cables to perform a remote laparoscopic cholecystectomy on a 68-year-old female patient [12]. In recent years, the rapid development of 5G communication technology and surgical robots in China has provided hardware support for remote surgery and offered medical teams the opportunity to explore the application of remote surgery via 5G networks. Currently, the limited number of clinical case reports of remote surgery is concentrated primarily in the fields of hepatectomy and cholecystectomy [13], nephrectomy [14], and gastrectomy [15]. Further exploration is needed to determine whether 5G remote robotic surgery technologies can be applied in thyroid surgery, which requires high-precision surgical operations.

In the present study, we innovatively applied 5G remote technology to thyroid surgery and successfully performed radical thyroidectomy in seven patients with thyroid cancer. To the best of our knowledge, this is the first pilot study in China to report the application of 5G-based telerobotic radical thyroidectomy. Thyroid surgery is characterized by the precision of its technology and surgical skill, with high requirements for functional protection and thorough resection. In this study, we used a surgical robot system to provide the surgeon with a three-dimensional, high-definition view of the surgical field, facilitating improved identification of important tissue structures in the neck. The system’s wrist rotation surgical instruments and stable and continuous robotic arms are convenient for precise surgical operations, helping provide accurate functional protection for the recurrent laryngeal nerve, superior laryngeal nerve, parathyroid gland, and neck blood vessels. Compared to conventional robotic surgery, special attention should be paid to real-time communication with assistants during remote robotic surgery. The operation should be slower and more stable, and a “small step” approach should be adopted to avoid rapid and significant movement of the instruments [16]. During the surgery, the surgeon performed primary lesion resection and regional lymph node dissection in a standardized manner, ensuring a thorough operation. Simultaneously, more attention was paid to standardization and refinement during the operation. When encountering blood vessels, an ultrasound knife was used for coagulation and closure to ensure safe treatment. Hem-o-lock was used to clamp the superior blood vessels of the thyroid gland to avoid emergency situations of intraoperative bleeding. Intraoperative neural monitoring technology was applied to standardize the protection of the recurrent laryngeal, vagus, and superior laryngeal nerves. Fine membrane dissection and nanocarbon parathyroid negative imaging technology were used to accurately identify and protect the parathyroid, achieving both tumor cure and functional protection, and achieving the same effect as conventional robotic thyroid cancer surgery.

Mastery of surgical procedures and enhanced coordination among surgeons, assistants, nurses, and surgical instruments are crucial for ensuring the safety and smooth progression of surgery, thereby laying a solid foundation for the advancement of remote surgery [17]. In remote thyroid cancer surgery, we have established a remote thyroid robot surgery team, which includes surgeons, anesthesiologists, mobile and instrument nurses, engineers and technicians. This study suggests that doctors can conduct remote surgical simulation operations, familiarize themselves with remote surgical scenarios, adapt to remote communication between operators and assistants, adapt to the opening and closing movements of instruments during remote surgery, and adapt to switching between robotic arms.

In this study, the safety of robotic thyroid surgery at different distances from the main terminal was investigated. The results show that robotic remote thyroid surgery at different distances and primary terminal positions offers promising feasibility and safety and represents a highly important surgical technique. Long-distance remote surgery enables high-precision technology and high-quality medical resources to overcome geographical limitations, providing high-quality medical care to more thyroid patients in resource-scarce areas, such as remote mountainous regions and islands, thereby improving treatment efficiency. In the future, remote surgery between countries or continents can enable the sharing and exchange of global medical technologies.

Signal transmission delay is an obstacle to remote robotic surgery. These delays are mainly due to errors in network transmission and hardware system processing of signal compression and decompression [18]. A previous study reported that the acceptable network delay time for remote surgical procedures was within 300 ms and that the ideal delay time should be less than 200 ms [19]. In this study, seven cases of remote surgery were performed, and the total delay of each remote surgery met the above requirements. There were no obvious delays or adverse events, such as lag, network disconnection, or frame loss, during surgery. Network security during remote surgery cannot be ignored. The entire surgical process was monitored in real time for network attacks to ensure network security. To address unexpected situations caused by network technology, instrument equipment and other issues during the operation, senior surgeons experienced in robot thyroid surgery were stationed at the bedside to provide onsite protection for patients. In the case of network technology issues or other emergencies, they would immediately initiate bedside robot surgery to ensure the safety of the surgery, strengthen the training of local doctors in robot surgery, and provide support and guarantee the implementation of remote surgery.

Based on the preliminary findings of this study, the application of remote robotic surgery in invasive thyroid cancer is technically feasible but requires strict selection criteria. Although current surgical systems possess fine dissection capabilities, managing complex procedures such as extrathyroidal extension and lymph node dissection has extremely high requirements for network latency, surgeon experience, and team collaboration. Current major limiting factors include the lack of haptic feedback (increasing the risk of injury to critical structures such as the recurrent laryngeal nerve and trachea), challenges in timely decision-making during unexpected intraoperative situations (such as tumor invasion of the trachea or esophagus), and stringent requirements for network stability (latency must be controlled within ≤ 100 ms). Regarding future development, we recommend a phased expansion of indications (progressing from low-risk cases to central and lateral neck dissection), the integration of augmented reality navigation and haptic feedback technologies, and the exploration of a “remote surgeon + on-site senior surgeon” dual-surgeon collaborative model to further enhance surgical safety.

## 5. Conclusions

In this pilot study, 5G-based telerobotic radical thyroidectomy demonstrated promising feasibility and safety. With stable network transmission and clear surgical fields, surgeons were able to perform delicate dissections, effectively protecting critical structures such as the recurrent laryngeal nerves, parathyroid glands, and trachea. Compared with traditional robotic thyroid surgery, 5G telerobotic surgery can achieve comparable precision in tissue handling, offering a viable solution for overcoming geographical barriers and sharing high-quality medical resources with underserved regions. Further large-scale studies are warranted to validate these findings.

## Figures and Tables

**Table 1:** Surgical master–slave system location of participants.

Cases	Master (Location/Mobile or Fixed)	Slave (Location/Fixed)	Distance Between Two Places (km)	Network Topology
Case 1	4/Fixed (5G)	2/Fixed (Broadband)	2200	5G-Broadband/Figure 1C
Case 2	Mobile (5G)	3/Fixed (Broadband)	240	5G-Broadband/Figure 1C
Case 3	Mobile (5G)	3/Fixed (Broadband)	240	5G-Broadband/Figure 1C
Case 4	Mobile (5G)	1/Fixed (5G)	439	5G-5G/Figure 1A
Case 5	Mobile (5G)	1/Fixed (5G)	439	5G-5G/Figure 1A
Case 6	Mobile (5G)	1/Fixed (5G)	22	5G-5G/Figure 1A
Case 7	Mobile (5G)	1/Fixed (5G)	22	5G-5G/Figure 1A

1, The First Medical Center of Chinese PLA General Hospital. 2, The Third Medical Center of Chinese PLA General Hospital. 3, Weifang Hospital of Traditional Chinese Medicine. 4, Sun Yat sen Memorial Hospital Affiliated to Sun Yat sen University.

**Table 2:** Demographic and clinical characteristics of participants.

Cases	Gender	Age (Years)	Weight (kg)	Number of Nodules	Thyroid Dysfunction	ACR TI-RADS	Bethesda
Case 1	Female	41	67	2	NO	5	6
Case 2	Female	46	71	2	NO	4	6
Case 3	Female	36	58	1	NO	4	5
Case 4	Female	49	63	1	NO	5	6
Case 5	Female	48	56	1	NO	5	6
Case 6	Female	42	85	1	NO	4	6
Case 7	Female	46	56	4	NO	5	6

**Table 3:** Perioperative and postoperative pathological condition.

Surgery	Fixed	Move					
	Case 1	Case 2	Case 3	Case 4	Case 5	Case 6	Case 7
Operative time (min)	78	95	100	80	94	82	110
Blood loss (mL)	15	10	10	5	10	10	20
Intraoperative complications	–	–	–	–	–	–	–
Parathyroid hormone (pg/mL)	15.3	28.1	23.2	19.2	17.3	33.6	34.1
Postoperative hospital duration (days)	5	3	3	3	3	4	4
Number of tumors	3	1	1	1	1	1	1
Maximum diameter (cm)	1.4	0.6	0.3	0.4	1.0	0.4	0.8
Unilateral/Bilateral	Bilateral	(Right) Unilateral	(Left) Unilateral	(Right) Unilateral	(Left) Unilateral	(Right) Unilateral	Bilateral
Types of pathology	PTC	PTC	PTC	PTC	PTC	PTC	PTC
lymph node metastasis ratio	6/12	3/7	0/13	0/3	2/6	0/8	2/11

PTC, papillary thyroid carcinoma.

**Table 4:** Delay time for two types of network connections.

Delay Time	Fixed Point (5G Wireless Network + Dedicated NETWORK)	Mobile (5G Wireless Network + Regular Wired NETWORK)					
	Case 1	Case 2	Case 3	Case 4	Case 5	Case 6	Case 7
Average round-trip delay (ms), t1	39	61.9	61.8	53	53	38	38
The average delay time of the response of the robotic arm to control signals (ms), t2	80	60	60	60	60	60	60
The average delay time of image compression and decompression processing in the surgical robot host system (ms), t3	40	23	23	23	23	23	23
Total delay (ms), t	159	144.9	144.8	136	136	121	121

Total delay t = t1 + t2 + t3; ms, milliseconds.

## Data Availability

The data presented in this study are available on request from the corresponding author.

## Supplementary Materials

The following supporting information can be downloaded at: https://www.mdpi.com/article/10.3390/jcm15103591/s1, The supplementary material includes supplementary technical information: 1. Connection between two sites (remote and local) in each case; 2. The number of degrees of freedom in “multi-degree-of-freedom fine operation capabilities”; 3. A detailed explanation of the “low latency, high speed, wide connectivity, and high reliability” of 5G technology; 4. A detailed account on the data rate, latency, jitter and outage requirements of each of the data streams; 5. Explanation on the mobile platform.

## Abbreviations

The following abbreviations are used in this manuscript:

5G	Fifth-Generation
FNAC	Fine needle aspiration cytology
ACR TI-RADS	American College of Radiology Thyroid Imaging, Reporting and Data System
BABA	Bilateral Axillary Areolar Approach
VPN	Virtual Private Network
