# Supplementary material for: A Pilot Study of Telerobotic Radical Thyroidectomy for Thyroid Cancer Using a 5G Network
Source: J Clin Med. 2026 May 8;15(10):3591. doi: 10.3390/jcm15103591 (PMC13207149; doi:10.3390/jcm15103591)
Supplement: Supplementary file 1 [file jcm-15-03591-s001.zip › jcm-4253395-supplementary.pdf]

Supplementary technical information

1. Connection between two sites (remote and local) in each case

Communication between two points of the remote surgery system can be achieved through optical fiber dedicated lines or Internet communication. Whether it is optical fiber dedicated line communication or Internet communication, the access methods of the two points must be consistent. When connecting to a fiber optic dedicated line, both points must be connected to form a local area network and point-to-point communication, without the need for VPN server forwarding. There are various kinds of Internet dedicated line access, including 5G, wired broadband and WIFI networks, which can access the Internet, and achieve communication forwarding between the two places through VPN cloud servers at specific locations, including control signal and 3D endoscope image transmission. In order to ensure the reliability of communication, each endpoint has been equipped with wired broadband and 5G networks, forming a dual network backup. That is, when using the wired broadband network, the 5G network serves as the backup. When using 5G networks, wired broadband serves as a backup. When the main network is interrupted, the backup network switches to the main network and can resume remote surgery by reconnecting the system. This process does not exceed 5 minutes. In the present study, among the 7 surgeries, the first 3 cases used 5G-broadband network, and the last 4 cases used 5G-5G network, both of which used VPN servers (please refer to the corresponding network topology diagram Figure 1 and Table 1).

By testing more than 400 free cloud server test network, looking for the lowest communication time delay between two points cloud server, and then buy and deployed on the VPN server, so as to realize the low latency of arbitrary points cost-effective transport. The control system and the image system can share a network access or use different network access methods. For instance, the control signal transmission is achieved through optical fiber dedicated lines for point-to-point communication, while the 3D endoscope image system communicates via wired broadband and a VPN server. The conference system can currently only be transmitted through a server. If the fiber optic dedicated line access method is adopted, an additional hardware server needs to be deployed within the

fiber optic dedicated line network. If it is Internet access, the server located in Shanghai will be used for communication forwarding.

2. The number of degrees of freedom in “multi-degree-of-freedom fine operation capabilities”

It includes 2 to 3 working arms and 1 mirror holding arm. The endoscope arm is used to hold the laparoscopic objective lens during the operation. Compared with the traditional holding by the laparoscopic assistant, it can provide a more stable image and avoid the problem of unstable visual field caused by hand shaking due to fatigue of the traditional laparoscopic assistant during the operation. The working arm is used to perform various operations during surgery. It has 7 degrees of freedom, including up and down, forward and backward, and left and right movements of the arm joint, as well as left and right, rotation, opening and closing, and end joint bending of the mechanical hand, totaling 7 actions. It can rotate 360° along the vertical head axis and 2709 ° along the horizontal axis, with each joint's range of motion exceeding 90° per day. Surgeons can perform continuous movements such as up and down, left and right, and rotation by operating the handle and translating and transmitting the hand movements of surgeons to the instrument end of the mechanical arm through the computer, making it more flexible than the human hand. Beside the operating table, there can be a physician's assistant and a hand-brushing nurse. According to the surgeon's wishes, they are responsible for replacing the mechanical hand of the robot and can operate through the auxiliary hole to perform some tasks such as pulling and suction to assist the surgery.

3. A detailed explanation of the “low latency, high speed, wide connectivity, and high reliability” of 5G technology

The “low latency” feature of 5G is compared with the previous generation of mobile communication technologies (such as 4G LTE) and some non-cellular communication technologies (such as Wi-Fi and traditional industrial buses), mainly reflected in the significant optimization of air interface transmission latency and end-to-end (E2E) latency at two levels. The theoretical minimum value of 5G air interface delay is ≤ 1ms (in

URLLC scenarios), and the typical value is 4-5ms, which is 50% - 90% lower than that of 4G. In the URLLC scenario, the end-to-end latency of 5G can be reduced to within 10ms, a decrease of 80% to 90% compared to 4G, meeting the strict requirements of real-time control services (such as autonomous driving and remote surgery). The “high rate” of 5G refers to the theory of 1 GBPS uplink rate, 10 times that of 4G. In practical applications, the uplink rate of a single 5G card can reach 200Mbps, fully meeting the requirements of remote surgeries. The “wide connection” of 5G is a means of the processing capacity of the concurrent access and persistent connections, 10 times that of 4G. Second, its signal sensitivity is remarkable. The terminal’s ability to receive weak signals has been significantly enhanced. It can operate normally under signals with a power 10 times weaker than 4G, enabling coverage in remote areas such as basements. 5G of "high reliability" refers to the high reliability and end-to-end wireless link reliability. In the URLLC scenario, the goal is a transmission reliability of 99.999%, corresponding to a packet error rate (PER) of $\leq 10^{-5}$. Through coding enhancement, multi-antenna diversity and network slicing technology, as well as multi-link redundant transmission, the end-to-end reliability can reach 99.9999%.

4. A detailed account on the data rate, latency, jitter and outage requirements of each of the data streams

Network performance requirements are as follows: Latency ≤ 110 milliseconds, jitter ≤ 30 milliseconds, and packet loss ≤ 0.1%.

Dual endoscope video stream rate is 15 Mbps, one-way latency ≤ 95 milliseconds, jitter ≤ 30 milliseconds, and frame rate ≥ 30 FPS;

Motion control signal rateis 4 Mbps, one-way delay 115 milliseconds, jitter ≤ 30 milliseconds, and packet loss ≤ 0.1%;

Video streamingin operating room and vital signs rate is 5 Mbps, latency ≤ 200 milliseconds, jitter ≤ 30 milliseconds, packet loss ≤ 0.1%.

5. **Explanation on the mobile platform**

The master console of the surgical robot is fixed inside a movable vehicle platform, but the vehicle remains stationary during the surgical process. The vehicle mounted remote mobile surgical robot system can serve as both a remote master for performing remote surgeries and a slave for conducting robot surgical training. Cases 2-7 utilized remote control function of the master console. Detail surgical master-slave system location of participants and schematic diagram of remote robotic surgical system operation and network connection were shown in Table1 and Figure1 (Case 2 - case 3: 5G - Broadband; Case 4 - case 7: 5G - 5G). The mobile platform is shown below.

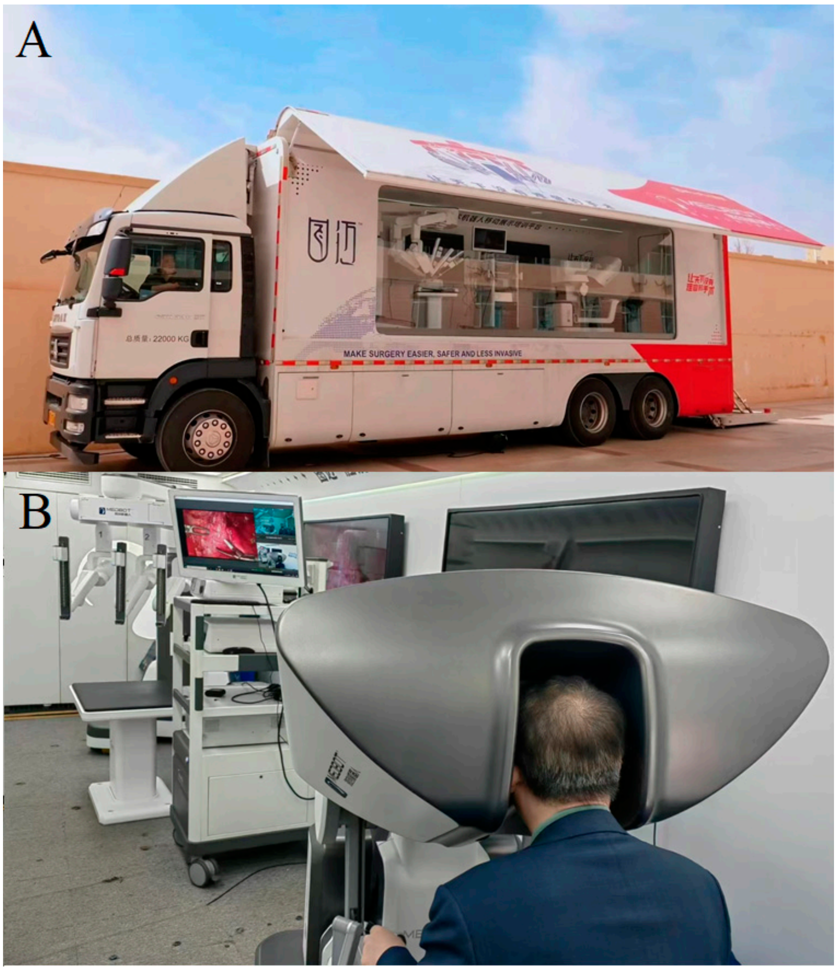

Figure S1 Mobile platform. A, Vehicle platform; B, Internal structure; the lead surgeon (men) operating the console (master system) to perform a remote robotic thyroidectomy on the patient.
